# A Saudi Child With Chronic Immune Thrombocytopenia and Vitiligo

**DOI:** 10.7759/cureus.9314

**Published:** 2020-07-21

**Authors:** Abdulqader Alhebshi, Hasanat Abbas, Hidayah M Alotaibi, Maryam Attaf, Arwa Al-Yamani

**Affiliations:** 1 Pediatric Oncology, Ministry of National Guard Health Affairs, Madina, SAU; 2 Pediatric, King Saud Bin Abdulaziz University for Health Sciences, Riyadh, SAU; 3 Pediatrics, Prince Mohammad Bin Abdulaziz Hospital, Ministry of National Guard Health Affairs, Medina, SAU; 4 Pediatric Hematology & Oncology, Princess Noorah Oncology Center, Ministry of National Guard Health Affairs, Jeddah, SAU

**Keywords:** tnf aip3 gene, tpo receptor agonist, citp, vitiligo, behcet

## Abstract

Chronic immune thrombocytopenia (ITP) is less commonly found in the children presenting with ITP. Patients usually present with petechiae, purpura, or active bleeding in the form of epistaxis or hematuria. The main aim of treatment in chronic ITP is to prevent major bleeding and to increase the platelet count. High doses of corticosteroids, intravenous immunoglobulin, rituximab, and eltrombopag, a thrombopoietin receptor agonist (TPO-RA), are medications that can be used. In this report, we present a case of chronic ITP in a 12-year-old child. In addition to features of chronic ITP, he also has vitiligo around his eyes and limbs. During treatment, he was resistant to steroids and did not respond to rituximab or eltrombopag. To understand the cause of his presenting features, we did multiple diagnostic evaluations. The whole-exome sequencing raises the possibility of auto-inflammatory syndrome Behcet-like (AISBL), which is a rare genetic disorder and not frequently reported in the available medical literature. AISBL is caused by mutations in the TNFAIP3 gene. According to our best knowledge, this is the first Saudi child diagnosed with chronic ITP and vitiligo with the possibility of AISBL that needs further genetic work-up to confirm the diagnosis.

## Introduction

Immune thrombocytopenia (ITP) is an immune-mediated acquired disease in adults or children characterized by a transient or persistent decrease of the platelet count and an increased risk of bleeding with time [1]. Patients present with isolated thrombocytopenia (less than 100 × 10^9^ cells/L) with no other predisposing conditions known to reduce the platelets. The condition is usually self-limiting and resolves with time, although some may persist for more than 12 months to become chronic ITP and affects 10% to 20% of children who present with ITP. Chronic ITP is more prevalent in older children. ITP affects approximately 1:10,000 children in the two- to six-year age group with an equal incidence in boys and girls [2]. The exact pathogenesis of ITP remains unknown. It is postulated that it is due to an auto-antibody formed against platelets, T cell mediated platelet destruction, and an impaired megakaryocyte function. The result is a markedly shortened life span of the circulating platelets to only a few hours, as they are rapidly cleared by the immune system [3]. Children with ITP usually present with a history of a preceding viral infection, which could possibly cause the generation of these auto-antibodies

The clinical presentation is petechiae and purpura, especially on the extensor surfaces, whereas some may present with buccal purpura and active bleeding in the form of epistaxis or hematuria. The complete blood count (CBC) is usually normal with only thrombocytopenia [4]. The main aim of treatment is to prevent major bleeding and raise platelet levels. High-dose corticosteroids, intravenous immunoglobulin (IVIG), rituximab, and the thrombopoietin receptor agonist (TPO-RA) are medications that can be used as needed. In patients not responding to these agents, either splenectomy is performed or immunosuppressive therapy is given [5].

In this case report, we present a rare case of chronic ITP and vitiligo that was suspected to have the auto-inflammatory syndrome, Behcet-like (AISBL).

## Case presentation

**Figure 1:**
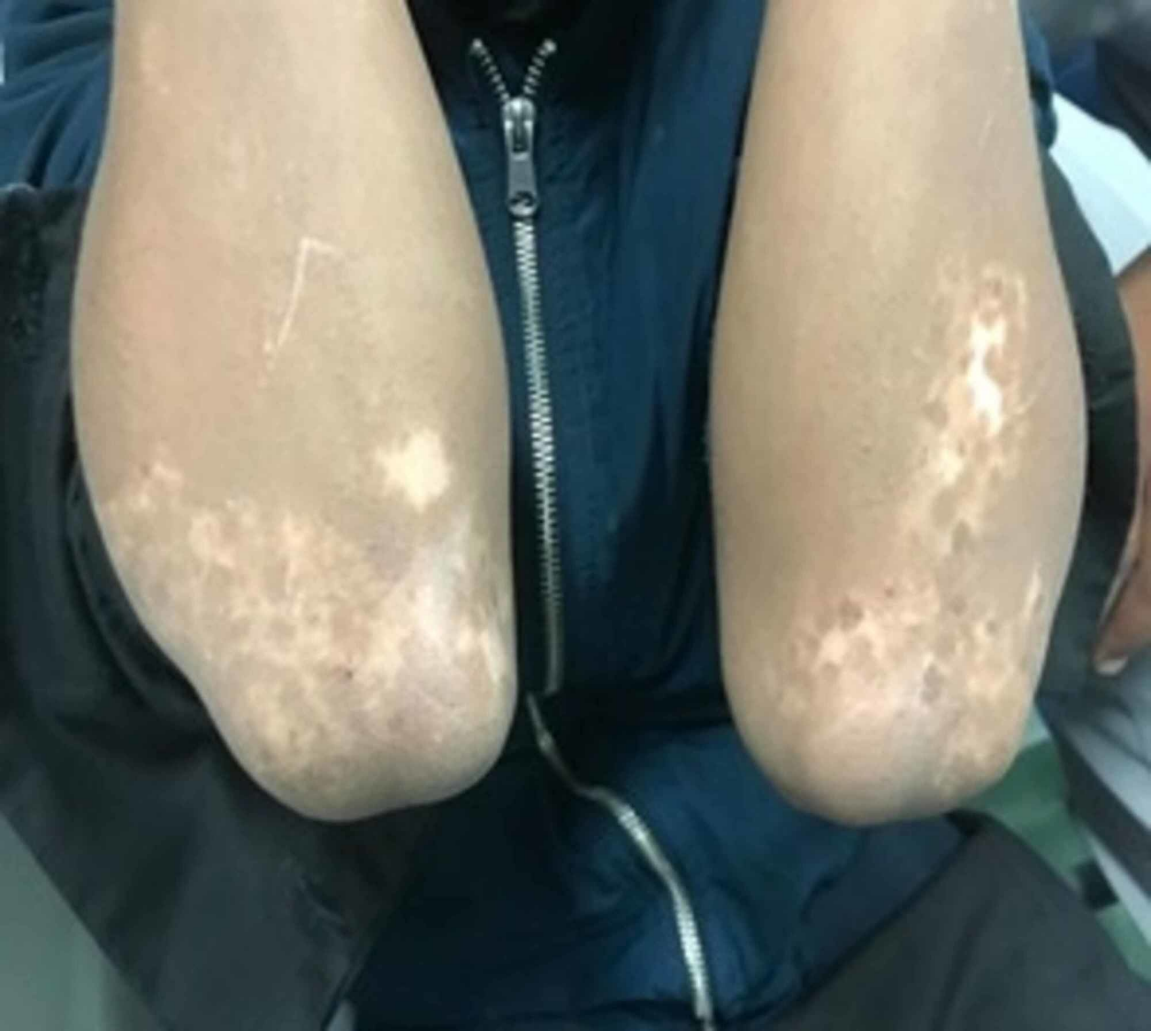
Hypopigmentation on the upper extremities, especially around the elbows

**Figure 2:**
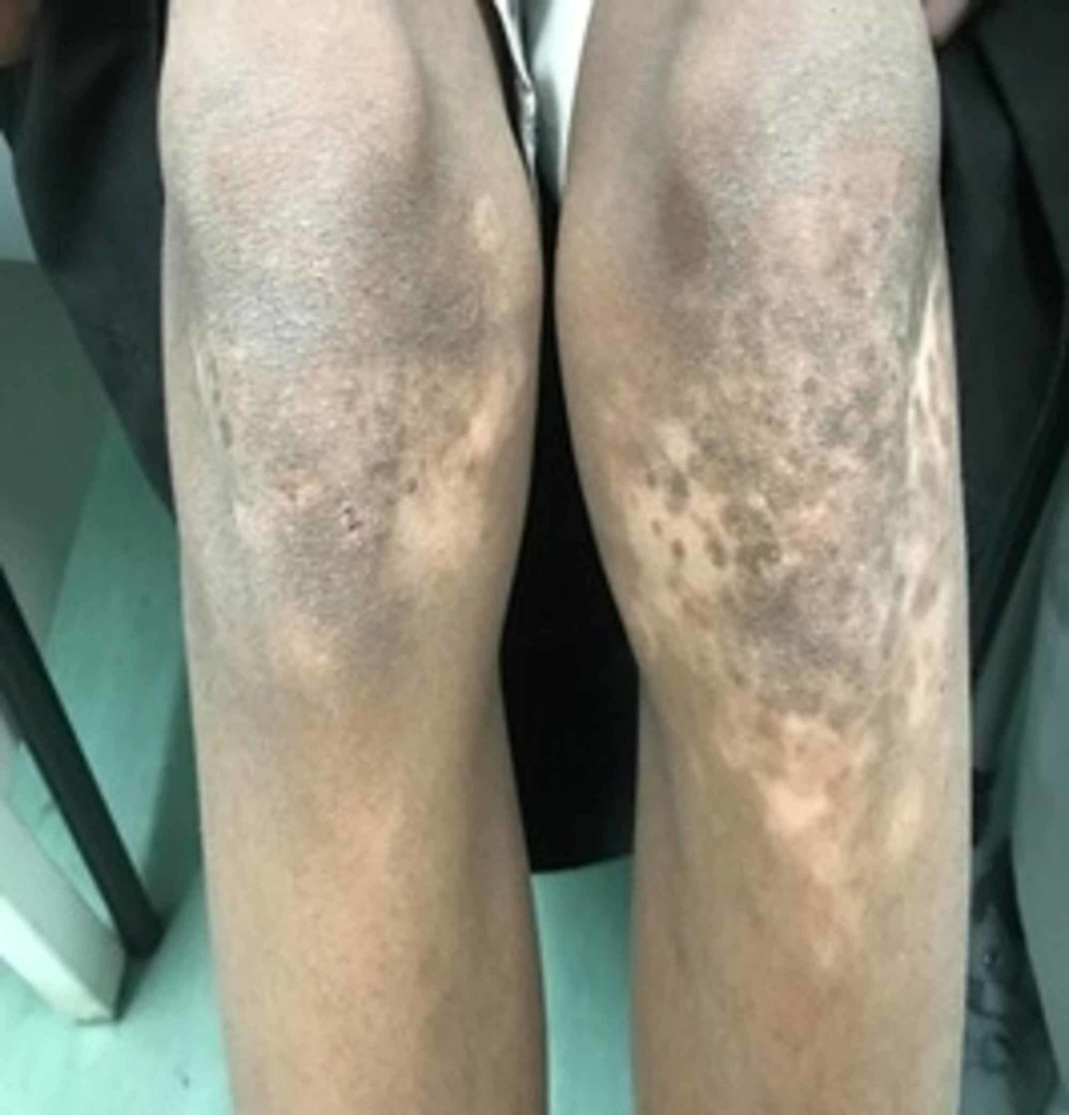
Hypopigmentation on the lower extremities, especially under the knees

A 12-year-old-boy presented for the first time in April 2017. He was diagnosed with ITP at the age of 9 years in a peripheral healthcare center. There was a history of mild epistaxis but no history of severe bleeding, trauma, or joint involvement. He received IVIG twice at the time of diagnosis. The lowest platelet count was 3 × 10^9^ cells/L (normal: 150-450 × 10^9^ cells/L); all other CBC indices were normal. The response was temporary. He had multiple hospitalizations due to recurrent epistaxis and ecchymosis. He is a product of non-consanguineous parents, has eight siblings, none of whom have any significant medical history. There was no family history of ITP, bleeding disorders, malignancies, or sudden deaths. On examination, he had ecchymosis and bruises on both legs. He also had depigmented patches around both eyes and on other parts of the body, such as both upper and lower extremities, especially the extensor surfaces, with varying sizes, white in color with sharp margins (Figures 1, 2), and there was hair involvement. No paleness or dysmorphic features were observed. There was no oral or genital ulceration, no organomegaly or lymphadenopathy, and his musculoskeletal system examination was normal. The patient was admitted to the hospital for further evaluation and management.

Diagnostic findings

Due to the multiple episodes of thrombocytopenia and resistance to first-line treatment, we performed other diagnostic procedures including the following:

1. A peripheral blood smear showed marked thrombocytopenia with large platelets, normal red blood cells, and white blood cell morphology.

2. A bone marrow biopsy revealed hypercellular bone marrow (90%), as seen with trilineage hematopoiesis, as well as an increase in megakaryocytes.

3. Flow cytometry indicated the normal expression of HLA-ABC and HLA-DR on the lymphocytes, which ruled out immunodeficiency. Level of alpha/beta double-negative T cell was normal.

4. The whole-exome sequencing (WES) (performed in Bioscientia Human Genetics) identified the heterozygous variant c.1420A>G (p.Thr474Ala) in exon7 of the TNFAIP3 gene (chr6:138200002), which leads to an amino acid exchange, and 10 of 16 bioinformatics in silico programs predict a pathogenic for this variant.

Therapeutic intervention

We initially treated the patient with IVIG (1 g/kg/day) for two consecutive days followed by oral prednisolone 2 mg/kg/day BID for two weeks and then tapered over 21 days. The patient did not show a significant increase in the platelet count, and the platelets only increased to 15 × 10^9^ cells/L. The patient was considered to be steroid-resistant, and the steroid was tapered over 21 days. The patient was given another dose of IVIG (1g/kg/day) for one day, which resulted in a rise in the platelet count. However, after one month, the platelet count again reduced to 4 × 10^9^ cells/L. As the patient is Rh-positive and the direct agglutinin test was negative, we gave him a single dose of Anti-D immunoglobulin (75 µg/kg IV for one dose), but with no response.

Subsequently, we prescribed rituximab 375 mg/m^2^ intravenous weekly for a total of four doses. However, the platelet count did not increase above 6 × 10^9^ cells/L even after the four doses. Another dose of IVIG was given, which resulted in the first significant increase in the platelet count to 107 × 10^9^ cells/L; however, after a few days, the platelet count dropped again with fluctuations. Finally, we prescribed eltrombopag 50 mg orally once daily in March 2018. The platelet count increased after a few days to 44 × 10^9^ cells/L for a couple of months and then it dropped again. For this reason, in September 2019, the dose was increased to 75 mg orally once daily (the maximum dose). The platelet count showed a significant improvement to the highest level since the diagnosis, 457 × 10^9^ cells/L for two months, and then it started to reduce again. Currently, the patient is on eltrombopag 75 mg once daily and clinically doing well, although his platelet count dropped again (baseline platelet count between 10 × 10^9^ cells/L and 15 × 10^9^ cells/L) and is considered as loss of response as per the International Working Group Criteria 2009 (criteria IWG). His school performance is good, and there is no history of bleeding episodes for a long time, even with physical activity.

For the hypopigmented patches, he was referred for dermatological consultation. The dermatologist diagnosed it as vitiligo and prescribed topical tacrolimus. Ophthalmology, pediatric-immunology, and pediatric-rheumatology consultations were done, and there were no significant findings.

## Discussion

The aim of treatment in a patient with ITP is to prevent episodes of bleeding, although it is rare in children. Our patient did not report any history of bleeding in the past. Severe bleeding manifestations are only seen when the platelet count falls below 10 × 109 cells/L [6]. It is important to monitor the platelet count during drug therapy to prevent such episodes. Our patient already received IVIG in the past and prednisolone was prescribed, but he was resistant to steroids. To rule out other causes of ITP, such as the autoimmune lymphoproliferative syndrome, we requested a flow cytometry, which was normal. Even though no bleeding was observed in our patient, his platelet counts were consistently below 10 × 109 cells/L. The next line of therapy was rituximab. Rituximab acts by binding to the CD20 antigen on the B cells and removes them from the circulation, slowing down the destruction of platelets [7]. Although there are no randomized clinical trials that evaluated the efficacy of rituximab in children with chronic ITP, a response of 61% has been reported in the literature [8]. The median time to respond was reported as three weeks in a review by Liang et al. [9]. However, our patient did not respond to rituximab after four weeks and was considered a treatment failure. Thrombopoietin (TPO) is an endogenous glycoprotein that regulates the production of platelets. TPO agonists, such as romiplostim and eltrombopag, have proven efficacy and are approved for use in adult ITP [10]. In children with chronic ITP, romiplostim had a variable response, but an interim analysis of a trial with eltrombopag (PEdiatric patients with Thrombocytopenia with ITP [PETIT]), has a good response [10]. Therefore, we prescribed eltrombopag. Since the patient had vitiligo, which was an unusual presentation, we requested WES. This revealed heterozygous variant c.1420A>G (p.Thr474Ala) in exon7 of the TNFAIP3 gene (chr6:138200002).

Behcet’s disease is a systemic inflammatory disease affecting young adults; it primarily involves the oral and genital mucosa, skin, and eyes, and is occasionally reported in children [11]. A similar familial Behcet-like auto-inflammatory syndrome has also been reported and is also known as autoinflammatory syndrome Behcet-like (AISBL). It has an autosomal dominant mode of inheritance and a similar manifestation as Behcet’s disease. Mutations in the TNFAIP3 gene, which regulate the NF-κB pathway, are involved in the causation of AISBL. Tumor necrosis factor-α-induced protein 3 (TNF/AIP3) is a protein with 790 amino acids coded by the TNFAIP3 gene located on chromosome 6. A loss of function mutation in the TNFAIP3 gene leads to haploinsufficiency of A20 (HA20), which causes an auto-inflammatory condition [12]. A study by Zhou et al. in 2016 described 14 cases of AISBL. These cases were from six unrelated families with various ethnic origins such as Turkish, European, American, and Dutch [13]. They confirmed the autosomal dominant inheritance. The patients described in this study were mostly female, and 12 of the 14 were 2 to 16 years of age. Our case is a male patient of Arabian origin, who was nine years old at the time of the first presentation. The clinical features reported by Zhou et al. were oral and genital ulcers, polyarthritis, skin rash, uveitis, fever, and hemolytic anemia, and only one case with idiopathic thrombocytopenia [13]. The features of the ulcers are consistent with Behcet’s syndrome. Our case did not report any such features. The initial presentation was chronic ITP with vitiligo, and AISBL was suspected by WES. Chronic ITP with Behcet’s syndrome is rarely reported in the literature. A case-report by Shin et al. reported a case of ITP with Behcet’s syndrome in a 33-year-old female of Korean origin [14], but this patient was a known case of Behcet’s syndrome and later diagnosed with ITP.

In contrast, our patient was a case of chronic ITP who suspected to have Behcet-like syndrome by WES and has revealed a heterozygous variant that has not been described in the literature so far as per The Human Gene Mutation Database (HGMD 2018.2) at the Institute of Medical Genetics in Cardiff, UK. Allele frequency of this variant in the general population has not been documented, and this is the first time detected in The Human Gene Mutation Database. Considering the available information, the variant is classified as a variant of uncertain significance. The pathogenic variants in the TNFAIP3 gene cause Behcet-like familial auto-inflammatory syndrome (OMIM: 616744) characterized by ulceration mucosal surfaces, particularly in the oral and genital areas. Additional more variable features include skin rash uveitis, polyarthritis, hemolytic anemia, and thrombocytopenia. Symptoms become apparent in the first or second decades of life. Taken together, it is unclear whether the detected heterozygous TNFAJP3 variant is causative for the phenotype of our patient.

The main limitation of this study is lack of segregation analysis test of the identified variant for parents and other family members to support WES finding. The test was not performed due to cost effectiveness.

## Conclusions

To the best of our knowledge, we report the first Saudi child who presented with chronic ITP and vitiligo. New heterozygous variant c.1420A>G was detected by WES that could be a causative variant of AISBL and has not been reported in the literature; therefore, it needs further gene test to confirm the finding. The best available treatment for chronic ITP should be considered to control patients' symptoms regardless of platelet count. We might need to perform genetic testing for all children with chronic or refractory ITP who lost their response to the second line of therapy to identify the underlying genotype and to support the diagnosis and treatment.
